# Radiation burden of assistant medical technicians at a medical accelerator

**DOI:** 10.4103/0971-6203.44480

**Published:** 2008

**Authors:** M. Gründel, F. Güthoff

**Affiliations:** Institute of Physical Chemistry, Georg August University, Tammannstr. 6, 37077 Goettingen

**Keywords:** Activation, CLINAC, dose rate, medical accelerator

## Abstract

A survey of a CLINAC 2100 C medical accelerator showed residual short half-life radiation after switching off the accelerator. This led to a dose of radiation for the medical employees when the patient was handled. The dose rate was measured with a dose rate meter FH40G, and annual dose for an assistant medical technician (AMT) was estimated under conservative conditions. In the assumed situation of 1000 patient treatment fractions with high-energy photons, an AMT would get an annual dose of 960 *μ*Sv, while the monthly dosimeter records would show zero, if the dose received is below threshold of 100 *μ*Sv.

## Introduction

Linear accelerators are often used in medical radiation therapy. For energies are greater than 8 MV, the linac output of photons is mixed with neutrons.[1] The neutrons result from prompt photodisintegration of high-energy photons interacting with the material of the accelerator head.[2] Specifically, the production of neutrons in the Varian Clinac 2100C/2300C has been discussed by Mao et al.[3] The patient dose of these neutrons was investigated in different surveys.[4] In 1977, Strandon[5] examined the influence of activation on the patient by these neutrons. The neutron dose of medical employees tends to be zero because neutrons exist only while the beam is on. During this time, no other person stays in the accelerator room except the patient. However, due to the activation of the accelerator head and the atmosphere[6] created by the neutrons, the employee is often exposed to enhanced radioactivity in its vicinity. Different studies[7,8] report the dose rate for technicians at a radiotherapy station while servicing the accelerator. A general consideration of the dose rate for different positions at the accelerator and different monitor units (MU) has been given by Wang *et al.*[9] and the results of a radiation protection survey at a medical accelerator have been presented by O'Brien.[10] The work of Rawlinson[11] focuses on the dose of a radiation therapist on a time scale of hours.

In this survey, in contrast to the one reported by Rawlinson, the dose rate of a CLINAC was measured on a time scale of a few minutes. This time interval is of interest for the estimation of the exposure of a medical employee. In routine operation, an assistant medical technician (AMT) enters the accelerator room directly after the beam stops for handling the patient or for the adjustment of a wedge. Therefore, the AMT works in the residual radiation field of the activated accelerator a few minutes after the beam has stopped.

To achieve adequate statistics for this investigation, measurements of the dose rate were done after a beam time of 60 s. These measurements were used to calculate the half-lives of the activation products. A beam time of 20 s was used to estimate the dose due to routine handling of a patient by the AMT.

## Materials and Methods

All measurements were done at a linear accelerator, CLINAC 2100C, from Varian. For an estimation of the upper radiation dose of a medical employee, measurements were done at the accelerator using a field size of 20 × 25 cm^2^[9] and a maximal photon beam energy of 20 MV; the output power was 3 Gy/min.

The dose rate, H*(10), was measured with a dose rate meter, FH40G, from ESM Thermo Eberline and recorded at intervals of 5 s. This recording time interval is appropriate for the observation of short time decay with the FH40G response time of 3 s.[12] The FH40 G has a proportional counter tube suitable for the entire energy range of 30 keV up to 1.3 MeV. Higher photon energies from the decay products should be negligible because the dose rate was registered at a distance of 70 cm from the isocenter. This is the region of the patient's upper body that is handled by the AMT. The measurements of radiation dose due to neutron activation were done directly after beam on time.

The measurements of the dose rate from the accelerator wedge were only started 15 s after the beam had stopped. This time interval was needed by the AMT to enter the accelerator room after the beam was stopped to take the wedge from the accelerator head and to place it in front of the dose rate meter at a distance of 10 cm.

A mobile γ-detector was not used because this survey focused on the dose and not on the determination of the activation products.[13]

## Results and Discussion

**Figure 1:**
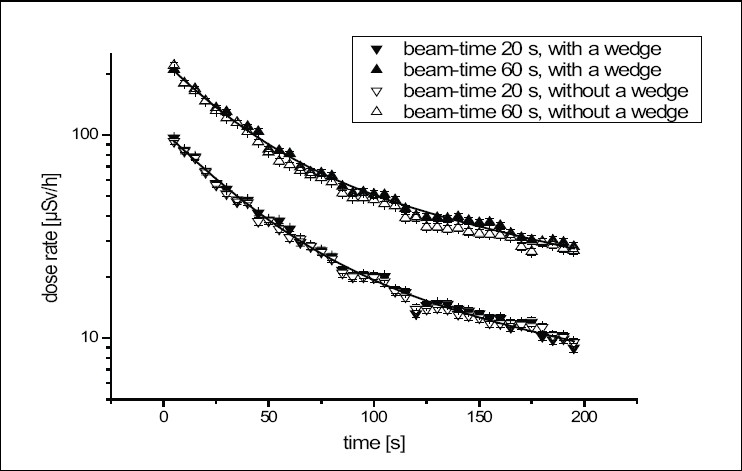
Time dependence of the dose rate (70 cm from isocenter); calculation with two exponential functions when using the wedge

The time dependence of the dose rate for four different situations is shown in Figure 1. Two curves show the progression of the dose rate after stopping the accelerator's beam without using a filter (for beam on times of 20 and 60 s). The other two curves refer to an experimental setup using a wedge of stainless steel in the beam.

In both cases, the dose rate rises with longer beam on times due to higher activation of the accelerator head components.[9] The decay characteristics of the dose rate are similar due to the fact that the configuration of the irradiated materials has not changed and due to the predominance of short-lived activation products in the decay for short irradiation times.

Measured values of the dose rate in Figure 1 can be described by two exponential functions: $Ḋ={Ḋ}_{0}+A\cdot \exp(-\ln2\cdot t{/}_{1}t_{1/2})+B\cdot \exp(-\ln2\cdot t{/}_{2}t_{1/2})$

Ḋ_0_ is the long-term background level of the dose rate. This level results from the long-lived activation products from repeated operation of the accelerator.[9] It was determined separately over a time interval of 5 min as 1 *μ*Sv/h. The half-lives of the dose rate decay were determined to _1_t_1/2_ = 132 (± 14 s) and _2_t_1/2_ = 21 (± 2 s). The 132 s agrees with the half-life of the ^28^Al (t_1/2_ = 134.76 s) which was found in previous investigations with other CLINACs.[11,13] Additionally, a half-life of 21 s was found in our measurements, which was not shown in the article of Rawlinson *et al.* The paper shows only longer half-lives of dose rates because their first measured value started one minute after stopping the beam.

An investigation of activity products[13] determined by gamma-spectroscopy revealed the absence of a product in the half-life region of 20 s. In this investigation,[13] the nuclides, ^26m^Al and ^203m^Pb with half-lives of 6.7 and 6.2 s respectively, were designated as short half-life candidates. Our measurements on the CLINAC did not support such short half-lives.

The half-life of 21 s could probably be due to the activation of silver from the electronics of the accelerator. The silver isotope, Ag-110, has a half-life of 24 s (^109^Ag (n, γ) ^110^Ag) and the determined decay time is close to this value. When silver is activated, a second silver isotope, ^108^Ag with a half-life of 144 s must be formed, which must be hidden by the ^28^Al. It may be noted that half life determination from dose rate measurements is not at all a sustainable method. In this survey, the focus was on the dose of the AMT and half-life determination should be considered in view of the main context of this presentation. Half-life measurements with gamma-spectroscopy should be used for the exact identification of activation products.

The following situation was postulated for the dose estimation of a medical employee: After a beam time of 20 s, an AMT approaches the patient 30 s after the beam stops; the handling of the patient needs 30 s. This results in a dose of:

(1) $$\begin{array}{ccc} E_{1} & = & H^{*}(10)\left[A_{1}\frac{{}_{1}t_{1/2}}{\ln2}\left[1-\exp\left(-\ln2\cdot \frac{t}{{}_{1}t_{1/2}}\right)\right]+A_{2}\frac{{}_{2}t_{1/2}}{\ln2}\left[1-\exp\left(-\ln2\cdot \frac{t}{{}_{2}t_{1/2}}\right)\right]\right]\\ & = & \mathrm{320 nSv} \end{array}$$

where A_1_ and A_2_ are prefactors from exponential fits of the dose rate and _1_t_1/2_ and _2_t_1/2_ are the determined half-lives of 132 s and 21 s. The dose rate 30 s after the beam has stopped is 55 *μ*Sv/h.

Based on this single exposure dose and taking into account that the AMT is exposed to radiation three times for an average time of 20 s while handling one patient, a medical employee with 1000 patient treatment fractions with high-energy photons per year will accumulate 960 *µ*Sv.

As a consequence of a common license due to a radiological protection ordinance, the personal dose needs to be measured by film badge dosimeters. These dosimeters are evaluated by an official radiation protection service on a monthly basis. Due to the short monitoring interval, all film dosimeters reported a zero dose below the detection limit of 0.1 mSv. As a result of this threshold value, the calculated value of 1 mSv per year is not recorded.

**Figure 2:**
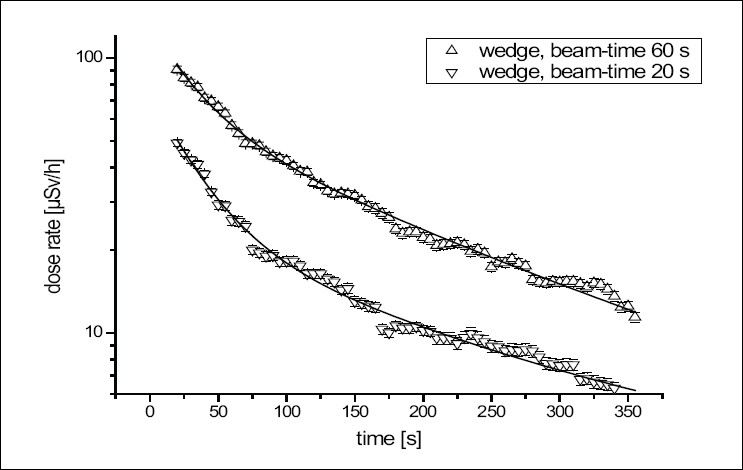
Time dependence of the dose rate of a wedge at a distance of 10 cm for different beam times (theoretical fit with two exponential functions)

Additional measurements were done directly on the stainless steel wedge which was manipulated during a radiation cycle. Figure 2 shows the time response of dose rate after a beam time of 20 or 60 s at a distance of 10 cm.

The dose rate can be described by the same exponential fit as shown before; half-lives of 23 (± 2 s) and 135 (± 12 s) were found. Again, it seems to be obvious that ^28^ Al is an activation product and the other product is the unknown material.

For a decrease of AMT exposure, the patient should be handled first followed by a manipulation on the wedge. The handling of the patient takes place at a larger distance from the accelerator head than does the wedge manipulation.

For the dose estimation for the AMT's hands while manipulating the wedge, two situations were evaluated: (a) the AMT changes the wedge 20 s after the beam stops, directly after entering the accelerator room, or (b) the change of the wedge is done 60 s after the beam stops and after handling the patient. In both cases, the manipulation of the wedge took 15 s.

Using equation (1), the estimated doses for the hands for both situations (a) and (b) are 181 and 96 nSv respectively. From 1000 uses in a year and two manipulations on the wedge, this leads to a dose of 362 (a) and 192 *µ*Sv (b) for the hands. With a tissue-weighting factor of 0.01 for the hands,[14] effective doses of 3.62 and 1.92 *µ*Sv are calculated.

These doses could be avoided by using a modern medical accelerator with enhanced dynamic wedge implementation.

## Conclusion

Exposure of an assistant medical technician from a medical accelerator was estimated from limited dose rate measurements. These involved the time-dependence of gamma dose rate of a CLINAC 2100 C from Varian at 70 cm from the isocenter and additionally, at 10 cm from the wedge. This dose rate results from the activation of the accelerator head and the wedge by photo-neutrons and depends on the beam-time and the activated materials.

With conservative estimates in the assumed patient situation of 1000 patient treatment fractions with high-energy photons, an AMT receives an annual dose of 960 *µ*Sv. It is reasonable to note the dose from additional manipulation of the wedge. This dose fraction can be avoided in modern accelerators with enhanced dynamic wedge implementations, or it can be reduced by a simple radiation protection instruction.

A problem seems to be the threshold of 0.1 mSv for standard film badge dosimeters because of which the dose recorded in every month is zero resulting in annual dose of 0 mSv, whereas the estimated dose lies approximately by 1 mSv. Due to the generally low dose received by staff in radiation oncology, a monitoring period of three months appears to be reasonable.
